# The effect of heparin concentration on results of venous blood gas of patients admitted to cardiac intensive care unit: A double-blind clinical trial

**DOI:** 10.37796/2211-8039.1242

**Published:** 2022-03-01

**Authors:** Rasool lakziyan, Fidan Shabani, Zohreh Sarchahi, Saeideh Mazloomzadeh, Fatemeh Shima Hadipourzadeh

**Affiliations:** a Rajaie Cardiovascular Medical and Research Center, Iran University of Medical Sciences, Tehran, Iran; b Department of Nursing, Faculty of Nursing, Neyshabur University of Medical Sciences, Neyshabur, Iran; c Cardiac Anesthesia Department, Rajaie Cardiovascular, Medical and Research Center, Iran University of Medical Sciences, Tehran, Iran

**Keywords:** Venous blood gases, Heparin concentration, Electrolytes

## Abstract

**Background:**

The aim of the present study was to investigate the effect of heparin (1000 IU/mL) in the blood sample on the results of venous blood gases of patients admitted to the cardiac intensive care unit.

**Materials and methods:**

The present double-blind randomized clinical trial study was performed on 282 samples from 141 patients admitted to the cardiac intensive care unit. Insulin syringes with heparin (1000 IU/mL) and heparin (5000 IU/mL) and 1 cc of blood sample were taken from the peripheral vein, then distributed in two syringes and given to the analyzer.

**Results:**

In the present study, the mean age of the samples was 49.96 ± 9.58. There was a statistically significant difference between the two groups in terms of values of partial pressure of carbon dioxide (PCO2) (P < 0.001), partial pressure of oxygen (PaO2) (P < 0.001), blood oxygen saturation (P < 0.001), bicarbonate ion (P < 0.001), excess base (P < 0.001), hemoglobin (P < 0.001), calcium (P < 0.001), potassium (P < 0.001), and sodium (P < 0.001) in the two groups.

**Conclusion:**

Overall, heparin (1000 IU/mL) led to a less disruption in the results of venous blood gases, and since it has not significantly increased the risk of clots, it is recommended to be used for venous blood gas sampling.

## 1. Introduction

Blood gas (BG) analysis is an essential part in diagnosing and managing changes in respiratory and metabolic parameters of critically ill patients and is an important tool for anesthesiologists and intensive care unit nurses [1]. Arterial blood gas (ABG) measurement is the most common test in intensive care units, but its blood sampling is invasive and painful and is associated with complications such as thrombosis, embolism, hematoma, aneurysm, distal ischemia, and infection [2–5]. It also causes needlestick injuries and subsequent exposure to the human immunodeficiency virus (HIV) and hepatitis among the medical staff [6]. When ABG measurement is difficult and technically impossible, the alternative method is to measure venous blood gases (VBG) [7].

The correctness of a BG test depends on the correct collection and analysis of blood samples, and laboratory errors account for most of medical errors [8]. However, in the case of VBG, many errors occur before analysis and when collecting blood samples that leads to incorrect concentrations of blood gases [9]. The efficiency of this diagnostic tool depends on the sampling accuracy, the syringe volume, the amount of blood sample, the type and amount of heparin, storage, and timing of sending the sample to interpret the results correctly. Although previous studies have shown that heparin may lead to errors in the blood gas processing, it is still considered the only suitable anticoagulant in BG analysis [10].

One of the most common practical problems related to VBG analysis is insufficient anticoagulation and the formation of small blood clots, which can lead to obstruction of the sampling path of analyzers and invalid results [11–14]. The minimum volume required for the analysis of BG parameters is 0.3 ml. Adding too much liquid heparin (sodium or calcium) to the blood sample can lead to positive bias by binding to positive ions and lead to negative bias by increasing the acidity level of the blood gases and the diluting blood sample [12, 15, 16]. Electrolytes and partial pressure of carbon dioxide (PCO2) seem to be dilution-sensitive variables, while the logarithm of the hydrogen ion concentration (pH) and the partial pressure of oxygen (PO2) are not significantly affected [17, 18]. Ionized calcium is more affected by the binding of heparin salts, although sodium and potassium may also be affected [19–21].

The Clinical and Laboratory Standards Institute (CLSI) and American Association for Respiratory Care (AARC) have published guidelines for the BG analysis that do not state the collection of blood in liquid heparinized syringes; however, they do warn the dilution and chemical effects of liquid heparin in BG tests [18, 22, 23]. Moreover, despite all these releases, non-standard BG sampling using liquid heparin (especially different sizes of syringes, needles and collecting different blood samples) is used to ensure patient comfort, it's easier use or unawareness of its effects [18, 24].

Considering the lack of standard protocols in reference books and different sampling methods (in terms of BG sample volume and heparin concentration for heparinizing syringes, different sizes of syringes and needles) and due to reduced combined effects and dilution in lower heparin concentration on BG results and comparing the incidence of clot formation in the two groups, the researcher decided to compare the effect of heparin (1000 IU/mL) and heparin (5000 IU/mL) on results of venous blood gas.

## 2. Materials and methods

The present double-blind randomized clinical trial was performed on 282 samples from 141 patients (2 samples from each patient) admitted to the intensive care unit (CCU) of 22 Bahman Hospital in Neyshabur in 2020. Inclusion criteria included: informed and written consent to participate in the study, CCU admission, and patients aged 18–60 years, hemoglobin concentration more than 10 mg/dl, included patients used anticoagulants, and antiplatelet.

After obtaining informed written consent from each of the research subjects, the eligible individuals were selected using convenience sampling method and sampling was continued until achieving the desired sample size. Data collection tools included demographic information form, intravenous blood gas results checklist, and blood gas test analyzer.

First, the demographic information form was completed by the researcher in the form of face-to-face interviews with the research subjects.

**Fig. 1:**
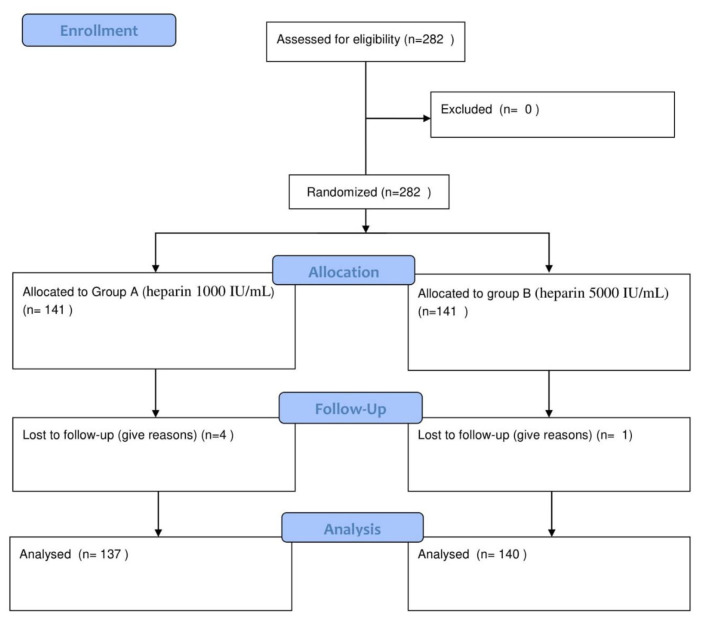
CONSORT 2010 flow diagram.

For intravenous sampling, insulin heparinized syringes were coded as A (heparin 1000 IU/mL) and B (heparin 5000 IU/mL) and the samples were numbered from 1 to 141 in each group. The reliability of insulin syringes was evaluated using ten syringes by filling and emptying them and measuring different concentrations of heparin remaining in the dead space of the syringe and needle. The samples were divided into two groups: 1000 IU/mL heparinized syringes and 5000 IU/mL heparinized syringes, which is the common heparinization method. Equal number of insulin syringes were heparinized by the researcher in one group (intervention group: A) with heparin 1000 IU/mL solution (to prepare heparin 1000 IU/mL, dilute one unit of heparin 1000 IU/mL with 4 cc of distilled water in a 5 cc syringe and increase its volume to 5 cc, each cc of which is equal to 1000 units), at the rate of 0.05 cc and with the same concentration of heparin 1000 IU/mL (0.05 cc) in the other group (control group: B). The syringe plunger was pulled back to the end once, so that their inner surface was heparinized. Then, the heparin was emptied once by pulling back the plunger forward. In order to maintain the same condition, two blood samples were taken from the peripheral venous line in terms of all possible confounding factors and homogenization of the two groups. In this regard, after taking 1 cc (100 units) of blood sample using a 100-unit syringe, equal blood samples (0.5 cc or 50 units per syringe) were divided in two heparinized insulin syringes: A (heparin 1000 IU/ml) and B (heparin 5000 IU/ml) and then placed on ice and sent to the laboratory within 10 minutes [10] along with the test sheet, in which the necessary parameters (patient demographic characteristics, body temperature, hemoglobin level, oxygen intake) were recorded. It should be noted that only the researcher knew about the nature syringe codes and grouping. Before sending the sample to the laboratory, attempts were made to ensure the absence of clots in the samples and if any clot was seen in the syringe, the results were recorded in the checklist to compare the two groups in terms of clot formation. Since clots cause damage to the device and the clot formation can be due to the use of inappropriate or insufficient anticoagulant or non-combination of heparin with the blood sample, therefore, the blood sample syringe was rolled or inverted between the palms of the sampler for 1 minute or 10 times before being given to the analyzer. Before analyzing the samples, the correct operation of the analyzer was confirmed by operator (calibration with three standard solutions of alkalosis, acidosis and normal, automatically during first 15 minutes in the morning and night shifts and manually after seeing each clot in the sample). The samples were given to a Medica Easy static analyzer (the USA) by a laboratory expert who did not know about heparinized syringes and codes A and B. It should be noted that the analysis time lasted for two consecutive minutes (syringes were placed randomly inside the device because there was a two-minute interval between their analysis). Demographic information of each patient was recorded in a questionnaire and checklist related to venous blood gas results. It should be noted that there were blood clots in four heparin (1000 IU/ml) syringes and one heparin (5000 IU/ml) syringe Fig. 1.

### 2.1. Ethical consideration

The present study was registered in the Iranian Registry of Clinical Trials with the code (IRCT20200929048885N1) and was approved by the Ethics Committee of the Shaheed rajaei's Cardiovascular Center with the number (IR.RH-C. REC.1399.064). CONSORT checklist was use to report the study [25].

The collected data were analyzed using SPSS ver. 20 and descriptive statistics and paired t-test and Wilcoxon t-test. P-value ≤0.05 was considered as the significant level.

## 3. Results

The present study was carried out on 141 people, including 60 women (42.6%) and 81 men (57.4%) with a mean age of 49.96 ± 9.58 years and a body temperature of 37.1 ± 0.35. With regard to demographic characteristics, 111 (78.7%) of the subjects were married and 47 (33.3%) were housewives. Also, 32.6% of people were illiterate, 112 people (79.4%) had no smoking and 83 people (58.9%) had acute coronary syndrome. Past medical history includes 71 patients with hypertension, 53 patients with diabetes, 8 patients with hyperthyroidism, 89 patients with hyperlipidemia, 18 patients with renal failure and 6 patients on dialysis, 41 patients with angioplasty, 5 patients with coronary artery bypass graft surgery, 1 patient with surgery Brain tumor and 4 patients had cancer. Patients presenting with arrhythmias often had a history of hyperthyroidism and renal failure. The most prevalent type of ACS was NSTEMI (n = 54), the most prevalent type of arrhythmia was atrial fibrillation. The preexisting disease for pulmonary edema was CHF (Table 1).

The results of paired t-test showed no significant difference between the pH values of heparin (5000 IU/ml) samples and heparin (1000 IU/ml) samples (P = 0.98). The results of paired t-test also showed a significant difference between PCO2, oxygen saturation (SO2), bicarbonate ion (HCO3), base excess (BE), hemoglobin and electrolytes (sodium, potassium and calcium) of heparin (5000 IU/ml) samples and heparin (1000 IU/ml) samples (P < 0.001). In other words, levels of PCO2, bicarbonate ion (HCO3), base excess (BE), potassium and calcium were higher in the heparin (1000 IU/ml) group than the heparin (5000 IU/ml) group. Also, levels of PO2, blood oxygen saturation (SO2), hemoglobin, and sodium were higher in the heparin (5000 IU/ml) group than in the heparin (1000 IU/ml) group (Tables 2 and 3).

The results of McNemar test showed no significant difference between the formation of clots in heparin (5000 IU/mL) samples and heparin (1000 IU/mL) (P = 0.25). There was one clot case in heparin (5000 IU/mL) samples and four clot cases in heparin (1000 IU/mL) samples. There was a common clot sample between heparin (1000 IU/mL) and heparin (5000 IU/mL) samples, but the difference was not statistically significant. There was no difference between the heparin (5000 IU/mL) and heparin (1000 IU/mL) groups in terms of clotting percentage and the number of clots in the heparin (1000 IU/mL) group was more than heparin (5000 IU/mL) group (Table 4). Table 5 also shows the oxygen intake of patients.

## 4. Discussion

The results of the present study revealed all parameters were significant except hydrogen ion logarithm (PH) and clot formation.

Sample dilution is not a unique characteristic of liquid heparin, as adding each liquid of a different composition to the blood (e.g. normal saline) will have a diluting effect. The fact that values of variables were statistically significant in the two groups could not be attributed to the effect of heparin dilution because 0.5 cc of blood and 0.05 cc of heparin (1000 IU/mL) and heparin (5000 IU/mL) (same blood dilution about 10%) were used in both groups of this study.

The heparin concentration and dilution in this study can be considered as a factor of insignificance of the statistical results of the hydrogen ion logarithm (PH), which was consistent with the previous studies [10, 26, 27], but inconsistent with studies by Coppola [28], Ashutosh Kumar [29], and Gholami [30] that suggested increasing heparin concentration had a significant effect on the results of hydrogen ion logarithm (PH). Despite the acidity of the heparin solution, the logarithm of the blood hydrogen ion (PH) is not affected by up to 40% heparin dilution due to the blood buffering capacity. In the present study, as suggested by some studies, 1 mg/cc concentration (200 UI/cc) was used in both groups to prevent the false effect of heparin on the results of the hydrogen ion logarithm (PH) [28, 31].

The results of this study show that increasing the heparin syringe concentration leads to a decrease in the parameters of PCO2, bicarbonate ion (HCO3), base excess (BE), which is consistent with the study by Gholami et al., Zokaei et al., Ashutosh Kumar et al., and Coppola et al.[26, 28, 30] suggesting that increasing heparin concentration led to a decrease in bicarbonate ion (HCO3) and PCO2 parameters, but was not consistent with a study by Barabadi et al.[10] suggesting that increasing heparin concentration had a significant effect on PCO2 parameter. Similarly, Gholami et al., Zokaie et al., and Barabadi et al. showed that an increase in heparin concentration led to a decrease in BE parameter [10, 26, 30], which was consistent with the present study.

The effect of dilution and excess heparin concentration can reduce PCO2, which has the greatest effect. The concentration should be at least half the volume of different blood syringes, and all the heparin should be removed from the syringe [10, 32, 33]. In the present study, considering the control of dilution effects and confounding factors, the significance of the results of this parameter is probably due to higher heparin concentration in the heparin (5000 IU/mL) group. On the other hand, considering its acidic nature, excess heparin concentration reduces the metabolic parameters (HCO3 and BE) and misdiagnosis of metabolic acidosis and leads to misinterpretation and improper treatment, which has little to do with the patient's underlying problems [10]. Bicarbonate ion (HCO3) is one of the most important chemical buffers in the body, and buffers combine with hydrogen ions to maintain the acid-base balance of the body to reduce the acidic effect of heparin, thus reducing free bicarbonate ions. Since the most abundant BE in blood plasma are bicarbonate ions, so if more heparin concentration is used to heparinize the syringe, the BE percentage will also decrease [34, 35]. According to Coppola 's study, the results of PCO2 and bicarbonate ion (HCO3) are inversely related to the increase heparin concentration, and 10% dilution led to a significant reduction in PCO2 and bicarbonate ion (HCO3) [36, 37].

On the other hand, the present study showed that increasing heparin concentration increased the PO2 and SO2, which was consistent with the studies by Gholami et al. and Ashutosh Kumar et al. [29, 30], but it was not consistent with the studies by Zokaie et al., Coppola et al., Barabadi et al. [10, 26, 28] showing no significant difference in the results of PO2 and PCO2. Also, according to the present study, increasing the heparin concentration led an increase in hemoglobin level, which was consistent with the study by Coppola et al. [28] but inconsistent with the study by Zokaie [26], which showed that increasing heparin concentration led to a decrease in hemoglobin levels.

Heparin has a relative oxygen pressure of 150 mmHg, which with increasing heparin concentration, the PO2 also increases. On the other hand, increasing the heparin concentration and increasing PO2 increases the SO2 concentration [28]. In Hopper's study, an 18.8%, and 34.1% dilutions of blood sample with a PO2 of 100 mmHg resulted in a significant increase in PO2 [38].

The increase hemoglobin concentration is probably due to its buffering property, and buffers, hemoglobin as the most important one, play a role in maintaining the acid-base balance. . Also, considering its acidic nature, negatively-charged hemoglobin showed a stringer affinity with oxygen+2 molecules that hydrogen +1 ion. Therefore, it increases the blood oxygen saturation percentage [39]. According to Zokaie et al., increasing heparin concentration reduces hemoglobin level considering its the dilution effects [26], which is inconsistent with the present study.

According to the present study, increasing the heparin concentration led to a decrease in the potassium and calcium ions but an increase in the sodium ion, which was consistent with studies by Barabadi [10], but it was inconsistent with the study by Coppola [28].

The elevated sodium level is due to the sodium heparin salt and blood sodium (NaH2Po4) is blood plasma buffer combined with hydrogen ions to maintain acid-base balance in blood samples and forms (NaH2Po4) and sodium ions due to the nature of acidified the heparin concentration, resulting in an increase in blood sodium ion level [40].

According to the results of the study by Sandler et al., the sodium ion group in the heparin (5000 IU/mL) was less affected than heparin (1000 IU/mL), which is probably due to the higher sodium concentration added to the sample from liquid sodium heparin, and this error is exacerbated at higher liquid heparin concentrations [41]. Kumar Sahoo and Hooper, excess liquid heparin due to its dilution and combination effects leads to a decrease in the measurement of positively charged ions such as potassium, calcium, and, on the other hand, there is almost no potassium in liquid sodium heparin and dilution of heparinized blood samples can lead to a significant reduction in potassium levels [18, 38]. In general, the liquid heparin concentration should not exceed 10 units per milliliter of blood, unless specialized heparin is used to eliminate the effect of heparin-binding activity of calcium [31].

The most important limitation of the present study was the low sample size of the number of participants, which limits the ability to generalize the results of the present study. It is better to conduct future studies on a larger sample size.

## 5. Conclusion

The results of the present study show a significant difference between two types of heparins groups in terms of the results of all parameters. Overall, heparin (1000 IU/mL) led to a less disruption in the results of venous blood gases, and since it has not significantly increased the risk of clots, it is recommended to be used for venous blood gas sampling.

## Supplementary Information



## Figures and Tables

**Table 1:** Frequency distribution of studied demographic and clinical variables.

Variable		Number	Percentage	Total number (percentage)
Sex	Male	81	57.4	141 (100)
	Female	60	42.6	
Level of education	Illiterate	46	32.6	141 (100)
	High school	28	19.9	
	Diploma	31	22	
	University	36	25.5	
Job	Employee	25	17.7	141 (100)
	Self-employed	37	26.2	
	Unemployed	23	16.3	
	Housewife	47	33.3	
	Student	9	6.4	
Marital status	Single	12	8.5	141 (100)
	Married	111	78.7	
	Divorced	4	2.8	
	Deceased spouse	14	9.9	
Smoking	Yes	29	20.6	141 (100)
	No	112	79.4	
Past Medical History	Hypertension	71	50.3%	141 (100)
	Diabetes	53	37.5%	
	Hyperlipidemia	89	63.1%	
	Hyperthyroidism	8	5.6%	
	renal failure and patients on dialysis	24	17%	
	Angioplasty	41	29%	
	CABG	5	3.54%	
	surgery Brain tumor	1	0.7%	
	cancer	4	2.83%	
	No past medical history	35	24.8	
Diagnosis	Acute coronary syndrome	83	58.9	141 (100)
	NSTEMI	45	54.3	
	STEMI	20	24	
	unstable angina	18	21.7	
	Heart failure	22	15.6	
	Pulmonary edema	14	9.9	
	Arrhythmia	22	15.6	
	(Atrial fibrillation	10	45.4	
	Atrial flutter	5	22.7	
	supraventricular tachycardia)	7	31.8	

**Table 2:** Comparison of the mean results of venous blood gases in heparin (1000 IU/mL) and heparin (5000 IU/mL) samples.

Variable	Heparin (1000 IU/mL)	Heparin (5000 IU/mL)	Heparin (1000 IU/mL)		Heparin (5000 IU/mL)		Test result	test statistics
				
	Median (Quartile range)/Mean ± SD	Median (Quartile range)/Mean ± SD	min	Max	min	max	P-value	
Hydrogen ion logarithm	(7.35–7.43)7.40	(7.36–7.43)7.40	3.371	7.54	3.369	7.964	0.98	−0.02
PCO2	(37.75–45.70) 42.20	(37.75–45.70) 42.	23.5	101.9	23.4	99.3	<0.001	3.85
Bicarbonate ion	(24.35–28.40)26.10	(24–28.50)25.90	14.2	39.2	3.3	39.2	<0.001	2.37
Base excess	(−0.75–2.25)	1 (−1.6–1.6)0.3	−5.2	5.9	−5.6	5.2	<0.001	12.81
Percentage of venous blood oxygen saturation	14.31 ± 63.29	14.30 ± 66.51	23.1	94.4	24.2	94.4	<0.001	−9.85
partial pressure of venous oxygen	10.90 ± 34.40	11.17 ± 35.70	15	70	15	74	<0.001	−10.27

**Table 3:** Comparison of mean electrolytes and hemoglobin levels in heparin (1000 IU/mL) and heparin (5000 IU/mL) samples.

Variable	Heparin (1000 IU/mL)	Heparin (5000 IU/mL)	Heparin (1000 IU/mL)		Heparin (5000 IU/mL)		Test result		
				
	Median (Quartile range)/Standard deviation ± mean	Median (Quartile range)/Standard deviation ± mean	minimum	maximum	minimum	maximum	P-value	Degree of freedom	Test statistics
Sodium	4.38 ± 136.75	5 ± 140.4	126.5	150.4	14.6	150.6	<0.001	135	−2.28
potassium	0.75 ± 4.12	0.70 ± 3.71	2.9	7.8	2.36	7	<0.001	135	15.67
Calcium	0.28 ± 1.28	0.22 ± 1	0.89	1.99	0.41	1.83	<0.001	135	15.95
hemoglobin	2.38 ± 14.66	2.28 ± 15.63	8.7	21.3	8	21.3	<0.001	134	10.18

**Table 4:** Frequency distribution of clot formation in heparin (1000 IU/mL) and heparin (5000 IU/mL) groups.

Variable	Clot formation in heparin (5000 IU/mL)			

		Yes (number/percentage)	No (number/percentage)	Total
Clot formation in heparin (1000 IU/mL)	Yes(number/percentage)	1(0.71)	3(2.13)	4(2.84)
	No(number/percentage)	1(0.71)	136(96.45)	137(97.16)
	Total	2(1.42)	139(98.58)	141(100)
	Yes(number/percentage)	1(0.71)	3(2.13)	4(2.84)

P = 0.25.

**Table 5:** Oxygen intake of patients.

Variable	Concentration of oxygen	Percentage	
The fraction of inspired oxygen (O_2_)	21%	88	140(99.3)
	21–40%	8.52	
	>40%	2.84	
