# Supplementary material for: The effect of heparin concentration on results of venous blood gas of patients admitted to cardiac intensive care unit: A double-blind clinical trial
Source: Biomedicine (Taipei). 2022 Mar 1;12(1):8–15. doi: 10.37796/2211-8039.1242 (PMC9236714; doi:10.37796/2211-8039.1242)
Supplement: Supplementary file 1 [file bmed-12-01-008-s001.docx]

**Title page**

**The Effect of Heparin Concentration of 1000 units per ml in Blood Samples on Results of Venous Blood Gas of Patients admitted to Cardiac Intensive Care Unit: A double-blind clinical Trial**

**Rasool lakziyan^1^, Fidan Shabani^2^, Zohreh Sarchahi^3^, Saeideh Mazloomzadeh ^4^, Fatemeh Shima Hadipourzadeh^5^***

1.MSc Student of Critical Care Nursing, Rajaie Cardiovascular Medical and Research Center, Iran University of Medical Sciences, Tehran, Iran.

2.Assistant Professor of Nursing, Rajaie Cardiovascular Medical and Research Center, Iran University of Medical Sciences, Tehran, Iran.

3.MSc of medical Surgical Nursing, department of Nursing, Faculty of Nursing ,Neyshabur University of Medical Sciences, Neyshabur, Iran.

4. Professor of Epidemiology, Rajaie Cardiovascular Medical and Research Center, Iran University of Medical Sciences, Tehran, Iran.

5*.Fellows of Cardiac Anesthesia, Cardiac Anesthesia Department ,Rajaie Cardiovascular, Medical and Research Center, Iran University of Medical Sciences, Tehran, Iran.

***Corresponding Author:** Fatemeh Shima Hadipourzadeh, Cardiac Anesthesia Department ,Rajaie Cardiovascular, Medical and Research Center, Iran University of Medical Sciences, Tehran, Iran.

**Email:** alianasiriu@gmail.com
